# Compliance to direct oral anticoagulation therapy and clinical outcomes after catheter ablation for atrial fibrillation: a nationwide cohort study

**DOI:** 10.1093/europace/euag151

**Published:** 2026-06-30

**Authors:** Bjarke Sihm Stender, Victor Kjær Eskildsen, Anders Kramer, Maja Hellfritzsch Poulsen, Kasper Korsholm, Kasper Bonnesen, Morten Schmidt, Mads Brix Kronborg, Jens Erik Nielsen-Kudsk

**Affiliations:** Department of Cardiology, Aarhus University Hospital, Palle Juul-Jensens Boulevard 99, Aarhus N, Aarhus DK-8200, Denmark; Department of Cardiology, Aarhus University Hospital, Palle Juul-Jensens Boulevard 99, Aarhus N, Aarhus DK-8200, Denmark; Department of Cardiology, Aarhus University Hospital, Palle Juul-Jensens Boulevard 99, Aarhus N, Aarhus DK-8200, Denmark; Department of Cardiology, Aarhus University Hospital, Palle Juul-Jensens Boulevard 99, Aarhus N, Aarhus DK-8200, Denmark; Department of Cardiology, Regional Hospital Gødstrup, Herning, Denmark; Department of Cardiology, Aarhus University Hospital, Palle Juul-Jensens Boulevard 99, Aarhus N, Aarhus DK-8200, Denmark; Department of Cardiology, Regional Hospital Gødstrup, Herning, Denmark; Department of Clinical Pharmacology, Copenhagen University Hospital – Bispebjerg and Frederiksberg, Copenhagen, Denmark; Department of Cardiology, Regional Hospital Gødstrup, Herning, Denmark; Department of Cardiology, Aarhus University Hospital, Palle Juul-Jensens Boulevard 99, Aarhus N, Aarhus DK-8200, Denmark; Department of Cardiology, Aarhus University Hospital, Palle Juul-Jensens Boulevard 99, Aarhus N, Aarhus DK-8200, Denmark

**Keywords:** Atrial fibrillation, Pulmonary vein isolation, DOAC, Compliance, Adherence, Stroke-prevention

## Abstract

**Background and aims:**

Life-long direct oral anticoagulant (DOAC) therapy is recommended after catheter ablation for atrial fibrillation (AF) in high-risk patients (CHA_2_DS_2_-VA≥2). Long-term DOAC compliance is crucial for effective stroke prevention. This study seeks to evaluate long-term compliance measured by adherence and persistence to DOAC therapy following first-time catheter abltation for AF.

**Methods and results:**

All Danish patients undergoing first-time catheter ablation between 2017 and 2024 were identified through Danish registries. Patients were stratified by CHA_2_DS_2_-VA score 0, 1, and ≥2. Primary outcomes were adherence and persistence to DOAC therapy at 1, 2, and 3 years after first-time catheter ablation. Adherence was defined as the proportion of days covered (PDC) ≥80%. Persistence was assessed as the proportion of patients covered based on the most recent pharmacy redemption before specific time points, given a 20% grace period. A total of 13 438 patients (32.1% female) were included. At 3 years post-ablation, the proportion of adherent patients with CHA_2_DS_2_-VA ≥2 (*n* = 7322) was 87.6% [95% cinfidence interval (CI): 86.8–88.4]. Applying a sensitivity analysis with a PDC ≥90% threshold, the proportion was 78.8% (95% CI: 77.8–79.8%). Persistence was 73.0% (95% CI: 71.6–74.4%) at 3 years. Rates of thromboembolic outcomes were low with a total incidence rate of the combined outcome of ischaemic stroke, transient ischaemic attack and systemic embolism at 6.6 per 1000 person-years (95% CI: 5.8–7.4). The incidence of major bleeding was found at 7.1 per 1000 person-years (95% CI: 6.3–8.0) for the combined cohort.

**Conclusion:**

While adherence to DOAC therapy was acceptable in patients with CHA_2_DS_2_-VA≥2, persistence declined over time and more than 20% were non-persistent by 3 years. Efforts to improve effective long-term stroke prevention may be warranted.

**Unstructured abstract:**

Life-long direct oral anticoagulant (DOAC) therapy is recommended after catheter ablation for atrial fibrillation in high-risk patients (CHA_2_DS_2_-VA≥2). Adherence and persistence to DOAC is crucial for effective stroke prevention.

Danish patients undergoing first-time catheter ablation between 2017 and 2024 were identified through registries.

A total of 13 438 patients were included. At 3 years post-ablation, the proportion of adherent and persistent patients with CHA_2_DS_2_-VA ≥2 (*n* = 7322) was 87.6% and 73.0%, respectively.

Adherence to DOAC was acceptable in patients with CHA_2_DS_2_-VA≥2. More than 20% of patients were non-persistent by 3 years. Efforts to improve effective long-term stroke prevention may be warranted.

## Graphical Abstract

**Graphical Abstract:**
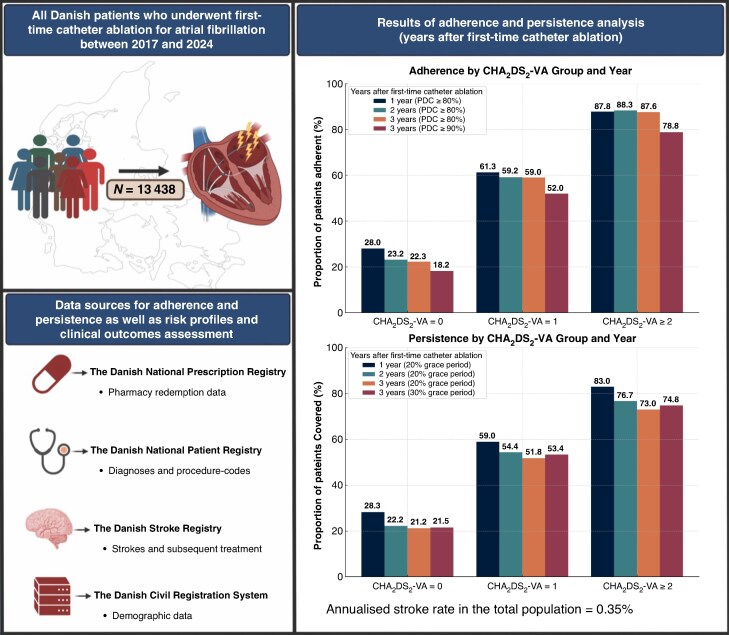
A nation-wide cohort study on adherence and persistence to direct oral anticoagulation following first-time catheter ablation for atrial fibrillation.

## Introduction

Atrial fibrillation (AF) is associated with significant morbidity and mortality.^1^ Pulmonary vein isolation (PVI) by catheter ablation is widely used for rhythm control in symptomatic patients, with proven reduction in AF burden and improvement in quality of life.^2^ However, the risk of AF recurrence warrants continued long-term anticoagulation therapy in patients with an estimated elevated thromboembolic risk.^3,4,5^

Direct oral anticoagulants (DOACs) constitute first-line stroke preventive therapy.^6–9^ Compliance, encompassing both adherence and persistence, to oral anticoagulation is critical for effective stroke prevention.^10–13^ Adherence refers to whether the patient takes the treatment as prescribed, while persistence refers to whether a patient continues on the treatment after initiation. While several studies have evaluated adherence and persistence in broader AF populations, results have varied widely across regions and healthcare systems, reflecting differences in study populations and methodology.^11,14–18^ However, data on long-term adherence and persistence following catheter ablation remain limited. Insights into adherence and persistence patterns may support post-ablation care and patient education, thereby contributing to improved stroke prevention. Utilizing the nationwide Danish patient and prescription registries,^19–21^ this study investigated the 3-year adherence and persistence among patients undergoing first-time catheter ablation for AF.

## Methods

### Study population

Our study cohort included all patients with AF who underwent a first-time catheter ablation for AF, between 1 January 2017 and 28 February 2025.^22,23^ Patients who had undergone catheter or surgical left atrial appendage closure (LAAC) before first-time ablation were excluded.^23^ Patients were subcategorized into three groups according to CHA_2_DS_2_-VA score 0, 1, or ≥2, for grading thromboembolic risk and DOAC therapy indication in line with contemporary European guidelines (see Supplementary material online, *Table S1*).^4^

### Data sources

The Danish healthcare system offers tax-funded universal access to healthcare, along with partial reimbursement for most prescription medication.^19^ A unique Civil Personal Register number, assigned at birth or upon immigration, enables linkage of all Danish health registries at an individual level and long-term follow-up with accurate censoring at death or emigration.^24^

The Danish National Patient Registry (DNPR) was used to identify the study cohort, CHA_2_DS_2_-VA score, and comorbidities through International Classification of Diseases (ICD-10) diagnosis and procedural codes.^21^ The DNPR contains data on all in- and outpatient hospital and emergency department contacts, with nationwide coverage since 1978.^21^ The coding for AF and catheter ablation for AF has been validated within the DNPR, with positive predictive values (PPV) of 95%^22^ and 100%,^23^ respectively. Diagnosis codes used for the definition of CHA_2_DS_2_-VA are applied in Supplementary material online, *Table S1*, along with corresponding validated PPV ranging between 90–100% for the majority of variables.^25–28^ Bleeding events were identified through the DNPR, using validated coding with a PPV of 93% and 94% for intracranial haemorrhage and extracranial major bleeding, respectively.^29^

Stroke was collected through the Danish Stroke Registry, capturing all strokes across Denmark with nationwide coverage since 2003 and a validated PPV of 94% for ischaemic stroke.^30^

Prescription data were collected through the Danish National Prescription Registry, which has complete nationwide coverage since 1995.^20^ Coverage was calculated using DOAC type, dosage, tablets per package, and the number of packages collected at each redemption. The Danish Civil Registration System (complete nationwide coverage since 1968) was used to obtain demographic information.^24^

A complete list of ICD-10, NOMESCO, and ATC codes for outcomes and covariates, including PPVs for validated cardiovascular diagnoses, is provided in Supplementary material online, *Table S1*.

### Consent

In accordance with Danish law, informed consent or ethical approval is not required for registry-based studies.

### Adherence

**Figure 1:**
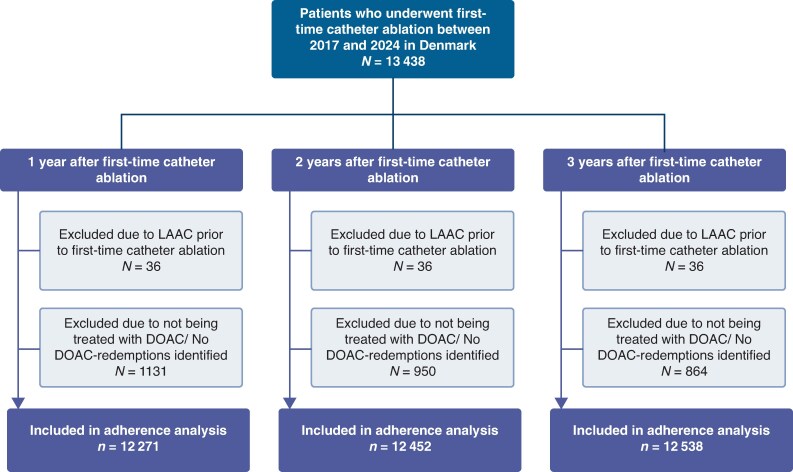
Inclusion and exclusion flowchart for adherence analysis. DOAC, direct oral anticoagulant; LAAC, left atrial appendage closure.

Adherence was assessed 1, 2, and 3 years after first-time catheter ablation using proportion of days covered (PDC).^18,31^ PDC was calculated by summation of coverage from all redeemed DOAC prescriptions in the given analysis period and patients with ≥80% coverage were considered adherent. A day was considered covered if the patient had sufficient medication from prior pharmacy redemptions to maintain continuous therapy on that day. Patients who died, switched to VKA, or underwent LAAC within the analysis period were censored from the time their DOAC indication ceased. Repeated ablation within the follow-up period was not regarded as a cause for censoring. A complete flowchart of inclusion in the adherence analysis can be seen in *Figure 1*.

### Persistence

**Figure 2:**
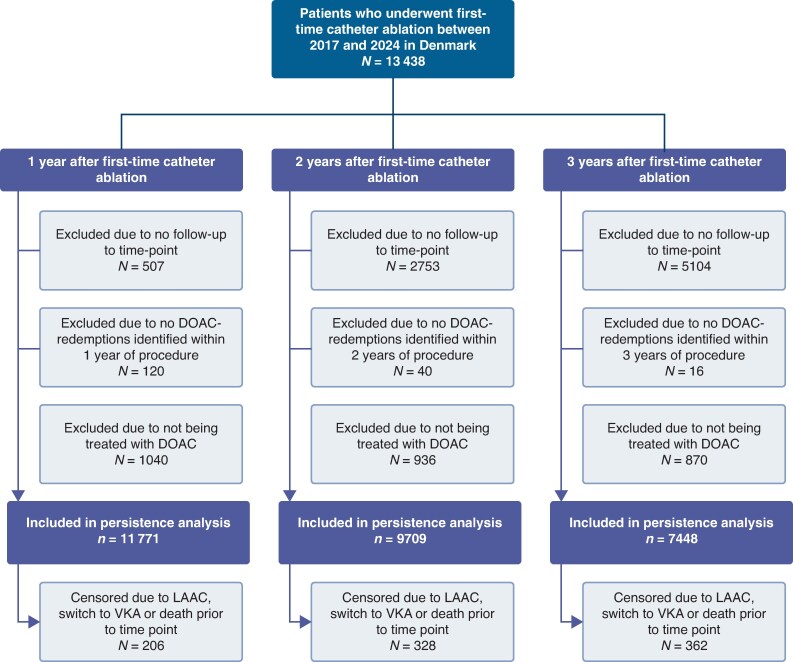
Inclusion and exclusion flowchart for persistence analysis. DOAC, direct oral anticoagulant; LAAC, left atrial appendage closure; VKA, vitamin K antagonist.

Patients were required to have redeemed at least one DOAC prescription within the first three years post-ablation to be eligible for persistence analysis. Persistence was evaluated using the proportion of patients covered method.^32^ At 1, 2, and 3 years after ablation, the most recent DOAC redemption before the time point was identified. Based on dosage, tablets per package, and the number of packages redeemed, including a 20% grace period, persistence was defined as a binary outcome, indicating whether the patient was covered at the time point. Patients who died, switched to VKA, or underwent LAAC before a given analysis time point were not classified as persistent or non-persistent; instead, they were censored. A complete flowchart of inclusion in the adherence analysis is shown in *Figure 2*.

### Study outcomes

The primary outcome was adherence and persistence to DOAC therapy following first-time catheter ablation, evaluated at 1-, 2-, and 3-year post-procedure. Secondary outcomes were a thromboembolic composite of ischaemic stroke, systemic embolism, transient ischaemic attack (TIA), along with the individual outcomes of ischaemic stroke, major bleeding, including intracranial haemorrhage and major extracranial bleeding defined by BARC≥2 (overt sign of haemorrhage requiring medical attention),^33^ and all-cause mortality.^34^

### Sensitivity analysis

We performed sensitivity analyses for both adherence and persistence. Recent studies indicated PDC ≥90% as an alternative cut-off for DOAC adherence to allow effective stroke prevention.^12,13^ Hence, a sensitivity analysis was performed using the 90% PDC threshold for adherence. For persistence, a sensitivity analysis was performed applying a more lenient grace period of 30% from the last redemption coverage.

### Statistical analysis

Index date and at-risk time were calculated from first registered date of first-time catheter ablation. Patients were followed until death, emigration or 3 years from index date. Baseline demographic data and risk factors were reported at index date while adherence, persistence and clinical outcomes were reported throughout the follow-up period. Persistence and adherence were reported as proportions with 95% confidence intervals (CIs), while baseline data are presented as numbers and percentages for categorical variables, and as medians and interquartile ranges for continuous variables. We reported the proportions of patients showing persistence to treatment at timepoint, non-persistent patients, and the proportion of patients having dropped out due to either death, having undergone LAAC, or a shift of treatment to VKA drugs during the period.^35^ Event rates for both composite and individual outcomes were expressed as unadjusted incidence rates per 1000 person-years with 95% CIs. All analyses were performed using STATA version 17 (STATA IC, StataCorp, College Station, TX).

## Results

### Study population

A total of 13 438 AF patients undergoing catheter ablation were included for analysis. The baseline CHA_2_DS_2_-VA-score was 0 in 13.2% of patients (*n* = 1769), 1 in 32.3% of patients (*n* = 4347), and ≥2 in 54.5% of patients (*n* = 7322). See *Table 1* for patient characteristics. Female sex accounted for larger proportions of the population in CHA_2_DS_2_-VA groups 1 and ≥2. In both groups 1 and ≥2, patients with prior strokes/TIA were categorized into group 2. Patients in these groups also had a higher mean HAS-BLED score.

**Table 1:** Characteristics by CHA_2_DS_2_-VA at time of AF ablation

	CHA_2_DS_2_-VA at time of AF ablation			
	0	1	≥2	Total
	*n* = 1769 (13.2%)	*n* = 4347 (32.3%)	*n* = 7322 (54.5%)	*n* = 13 438 (100%)
Sex				
Female	316 (17.9%)	1237 (28.5%)	2757 (37.7%)	4310 (32.1%)
Age at procedure*	58.2 (52.0–61.9)	61.0 (56.0–64.4)	70.0 (66.3–74.2)	66.8 (60.5–72.1)
CHA2DS2-VA	0.2 (0.4)	1.3 (0.5)	2.9 (0.9)	2.0 (1.3)
Congestive heart failure	0 (0.0%)	18 (0.4%)	1485 (20.7%)	1503 (11.3%)
Hypertension	0 (0.0%)	3518 (81.3%)	6977 (97.0%)	10 482 (79.0)
Stroke	0 (0.0%)	0 (0.0%)	85 (1.2%)	0 (0.0%)
Vascular disease	0 (0.0%)	25 (0.6%)	640 (8.9%)	665 (5.0%)
HAS-BLED	0.0 (0.2)	1.0 (0.3)	1.9 (0.5)	1.4 (0.8)
Kidney disease	—	14 (0.3%)	106 (1.5%)	—
Liver disease	—	9 (0.2%)	46 (0.6%)	—
Major extracranial bleeding		15 (0.3%)	44 (0.6%)	
Intracranial bleeding	0 (0.0%)	0 (0.0%)	0 (0.0%)	0 (0.0%)
Active antiplatelet therapy	72 (4.1%)	184 (4.2%)	376 (5.1%)	630 (4.7%)

*Age reported as median, (IQR).Remaining continuous variables reported as (mean, (SD), categorical variables reported as *n* (%). (− = omitted due to small numbers).

IQR, interquartile range; SD, standard deviation.

### Adherence to DOAC therapy

Among patients with CHA_2_DS_2_-VA ≥2, adherence defined by DOAC coverage ≥80% at 1-year follow-up was 87.8% (95% CI, 87.0–88.5). The median duration of follow-up in this period was 365 days (IQR, 365–365). At 2- and 3-year follow-up, adherence was 88.3% (95% CI, 87.5–89.1) and 87.6% (95% CI, 86.8–88.3) with 730 days (IQR, 616–730) and 1036 days (IQR, 619–1065) follow-up, respectively. The proportions of adherent patients were lower in the CHA_2_DS_2_-VA 0 and 1 groups at all timepoints (*Table 2*).

**Table 2:** Time-dependent proportion of adherence by CHA_2_DS_2_-VA

	CHA_2_DS_2_-VA at time of AF ablation		
Adherence to DOAC (PDC)	0	1	≥2
***Year 1***	28.0% (25.8–30.2)	61.3% (59.8–62.8)	87.8% (87.0–88.6)
***Year 2***	23.2% (21.2–25.3)	59.2% (57.7–60.8)	88.3% (87.5–89.0)
***Year 3***	22.3% (20.3–24.4)	59.0% (57.5–60.5)	87.6% (86.8–88.4)

Adherence defined as ≥80% DOAC coverage. All estimates reported as % (95% CI).

CI, confidence interval; DOAC, direct oral anticoagulation; PDC, proportions of days covered.

### Persistence to DOAC therapy

At 1 year, 83.0% (95% CI, 82.0–83.9) of patients with a CHA_2_DS_2_-VA-score of ≥2 remained persistent, while 15.2% (95% CI, 14.4–16.1) were non-persistent and 1.8% (95% CI, 1.5–2.2) were censored due to death, VKA switch, or LAAC. By 2 years follow-up, persistence declined to 76.7% (95% CI, 75.6–77.9), with a discontinuation rate of 19.4% (95% CI, 18.4–20.5), and 3.8% (95% CI, 3.3–4.4) were censored. At 3 years, 73.0% (95% CI, 71.5–74.4) remained persistent on DOAC therapy, while 21.2% (95% CI, 19.9–22.6) were non-persistent DOAC and 5.8% (95% CI, 5.1–6.6) were censored (*Figure 2*). The proportion of persistent patients was lower in the CHA_2_DS_2_-VA 0 and 1 groups at all time-points (*Table 3*).

**Table 3:** Time dependent proportion of persistence by CHA_2_DS_2_-VA

	CHA_2_DS_2_-VA at time of AF ablation		
Persistence to DOAC	0	1	≥2
***Year 1***			
* Persistent*	28.3% (26.2–30.6)	59.0% (57.4–60.5)	83.0% (82.0–83.9)
* Non-persistent*	70.1% (68.4–73.0)	40.0% (38.4–41.6)	15.2% (14.4–16.1)
* Censored*	1.0% (0.6–1.7)	1.0% (0.7–1.4)	1.8% (1.5–2.1)
***Year 2***			
* Persistent*	22.2% (19.2–24.6)	54.4% (52.7–56.2)	76.7% (75.6–77.9)
* Non-persistent*	76.3% (74.0–78.6)	43.9% (42.2–45.6)	19.4% (18.2–20.5)
* Censored*	1.4% (0.9–2.2)	1.7% (1.3–2.2)	3.9% (3.3–4.4)
***Year 3***			
* Persistent*	21.2% (18.7–23.8)	51.8% (49.9–53.7)	73.0% (71.6–74.4)
* Non-persistent*	77.2% (74.5–79.7)	45.8% (43.9–47.7)	21.2% (19.9–22.5)
* Censored*	1.6% (1.0–2.6)	2.5% (1.9–3.1)	5.8% (5.1–6.6)

Persistent: covered by latest dosage + 20% grace period at analysis timepoint. Non-persistent: not covered by a DOAC redemption at timepoint. Censored switch of therapy or death before timepoint. All estimates reported as % (95%CI).

CI, confidence interval; DOAC, direct oral anticoagulation; PPC, proportions of patients covered.

### Sensitivity analysis

When applying a 90% PDC threshold to define adherence, proportion of adherent patients at 3 years in CHA_2_DS_2_-VA groups 1 and ≥2, declined to 52.0% (95% CI, 50.4–53.5) and 78.8% (95% CI, 77.8–79.7), respectively.

For the sensitivity analysis of persistence, a more lenient 30% grace period on coverage from the latest redeemed prescription up to the analysis time point led to a modest increase in all estimates, but without significant changes (*Table 4*).

**Table 4:** Sensitivity analyses of adherence and persistence

	CHA_2_DS_2_-VA at time of AF ablation		
	0	1	≥2
	**Adherence to DOAC therapy:** *Defined by 90% PDC*		
***Year 1***	19.6% (17.7–21.7)	47.0% (45.4–48.6)	68.5% (67.3–69.6)
***Year 2***	18.7% (16.8–20.6)	51.7% (50.1–53.2)	78.2% (77.7–79.2)
***Year 3***	18.2% (16.4–20.2)	52.0% (50.4–53.5)	78.8% (77.8–79.7)
	**Persistence on DOAC therapy:** *Grace period set at 30%*		
***Year 1***			
* Persistent*	29.5% (27.3–31.9)	60.6% (59.0–62.1)	84.3% (83.4–85.2)
* Non-persistent*	69.4% (67.1–71.7)	38.4% (36.8–39.9)	13.9% (13.0–14.7)
* Censored*	1.0% (0.6–1.7)	1.0% (0.7–1.4)	1.8% (1.5–2.2)
***Year 2***			
* Persistent*	23.0% (20.8–25.4)	55.8% (54.1–57.5)	78.7% (77.5–79.8)
* Non-persistent*	75.5% (73.1–77.8)	42.5% (40.8–44.2)	17.4% (16.4%-18.5)
* Censored*	1.4% (0.9–2.2)	1.7% (1.3–2.2)	3.9% (3.3–4.4)
***Year 3***			
* Persistent*	21.5% (19.0–24.1)	53.4% (51.5–55.4)	74.8% (73.4–76.1)
* Non-persistent*	76.9% (74.2–79.4)	44.1% (42.2–46.1)	19.4% (18.2–20.7)
* Censored*	1.6% (1.0–2.6)	2.4% (1.9–3.1)	5.8% (5.1–6.6)

Adherence defined as ≥80% DOAC coverage. Persistent: covered by latest dosage + 20% grace period at analysis timepoint. Non-persistent: not covered by a DOAC redemption at timepoint. Censored: switch of therapy or death before timepoint. All estimates reported as % (95% CI).

CI, confidence interval; DOAC, direct oral anticoagulation; PDC, proportions of days covered; PPC, proportions of patients covered.

### Secondary outcomes

See *Table 5* for outcome results across all strata. In the total population, the composite thromboembolic outcome of ischaemic stroke, systemic embolisms, and TIA during the 3-year follow-up was observed as 6.6 per 1000 person-years (95% CI, 5.8–7.54). The incidence rate of ischaemic stroke was 3.5 per 1000 person-years (95% CI, 2.9–4.1). For the full cohort, 296 patients died during the 3-year follow-up, yielding an all-cause mortality rate of 7.6 per 1000 person-years (95% CI, 6.7–8.0). Major bleeding was observed as 7.1 per 1000 person-years (95% CI, 6.3–8.0).

**Table 5:** Incidence rates of adverse clinical events by CHA_2_DS_2_-VA group

	CHA_2_DS_2_-VA at time of AF ablation			
	0	1	2	Total
	*n* = 1769 (13.2%)	*n* = 4347 (32.3%)	*n* = 7322 (54.5%)	*n* = 13 438 (100%)
IS + SE + TIA	16 [3.1 (1.6–4.6)]	67 (5.3 (4.0–6.5))	175 [8.2 (7.0–9.4)]	258 [6.6 (5.8–7.4)]
IS	9 [1.7 (0.6–2.9)]	30 [2.4 (1.5–3.2)]	99 [4.6 (3.7–5.6)]	138 [3.5 (2.9–4.1)]
Major bleeding^a^	10 [1.9 (0.7–3.1)]	62 (4.9 [3.7–6.1)]	207 [9.7 (8.4–11.0)]	279 [7.1 (6.3–8.0)]
All-cause mortality	17 [3.3 (1.7–4.9)]	72 [5.7 (4.4–7.0)]	207 [9.7 (8,3–11.0)]	296 [7.6 (6.7–8.4)]

All estimates reported as *n* [incidence rate per 1000 person-years (95% CI)].

CI, confidence interval; IS, ischaemic stroke; SE, systemic embolism; TIA, transient ischaemic attack.

^a^Major extracranial bleeding + intracranial haemorrhage.

In patients with CHA_2_DS_2_-VA ≥2, the incidence rate of the composite thromboembolic outcome was 8.2 per 1000 person-years (95% CI, 7.0–9.4), compared with 5.3 (95% CI, 4.0–6.5) and 3.1 (95% CI, 1.6–4.6) in the CHA_2_DS_2_-VA 1 and 0 group, respectively. Within 3 years of follow-up, the incidence rate of ischaemic stroke was 4.6 per 1000 person-years (95% CI, 3.7–5.6) in the CHA_2_DS_2_-VA≥2 group, compared with an incidence rate of 2.4 (95% CI, 1.5–3.2) in the CHA_2_DS_2_-VA 1 group and 1.7 (95% CI, 0.6–2.9) in the CHA_2_DS_2_-VA 0 group.

## Discussion

In this nationwide cohort of patients undergoing first-time catheter ablation for AF, we found that DOAC adherence remained acceptable and persistence moderate for patients with a CHA_2_DS_2_-VA score of ≥2 with >85% of patients classified as adherent and 73% as persistent at 3 years. Around 50% of patients with CHA_2_DS_2_-VA 1 remained adherent and persistent to DOAC throughout the 3-year follow-up. The rates of ischaemic stroke and major bleeding were low. However, nearly one in four high-risk patients with DOAC indication demonstrated ≥10% of follow-up time without DOAC coverage. Moreover, treatment gaps were observed in approximately one in four patients with high comorbidity burden 3 years post-procedure. This suggests that meaningful gaps in anticoagulation remain common despite favourable aggregate adherence rates.

### Adherence and persistence

While prior studies have assessed DOAC adherence and persistence in general AF population,^14^ our study comprised younger patients with a lower mean CHA_2_DS_2_-VA score. Our cohort aligns with the typical profile of catheter ablation candidates seen in other procedural studies, and reflects a distinct, subgroup with a lower thromboembolic risk within the broader AF population.^36^ Females accounted for 32% of this catheter ablated cohort, though the female share of the general AF population has been estimated to 41%.^37^ This gender disparity is not unique to the Danish cohort and has been described in several studies and summarized in the comprehensive consensus statement on sex differences in cardiac arrythmias by the European Heart Rhythm Association.^38^

While DOACs remain first-line stroke prevention, the evidence remains sparse in the setting following successful AF ablation. The recent ALONE-AF trial provided supporting evidence of cessation of DOAC in patients without signs of AF after 1 year. However, the risk of AF recurrence was 10%, patients were at low ischaemic risk, and the main benefit was driven by reduction of bleeding events, while the study was not powered for ischaemic events. In the recently published OCEAN trial, patients were randomized between rivaroxaban and aspirin following catheter ablation. No clinical gain from rivaroxaban was detected, though thromboembolic event rates were lower than expected for both groups.^39^ The DESTINATION trials (NCT06615596) is still pending, and may provide further insights, while the OPTION trial, comparing LAAC against DOAC for stroke prevention after catheter ablation, provided supporting evidence of LAAC to maintain stroke prophylaxis while reducing bleeding risk in patients at high ischaemic risk.^40,41^

Continued efficacy of DOAC treatment relies on compliance, especially given the short half-life of these drugs.^17,42–44^ The adherence rate in our CHA_2_DS_2_-VA ≥2 cohort was notably higher than the 74% (95% CI, 68–79) reported at 1 year in a large meta-analysis including 500 000 unselected AF patients.^17^ In this nationwide registry cohort of AF patients who underwent catheter ablation, adherence appeared higher than in broader unselected AF populations; however, long-term persistence was suboptimal especially among those with high CHA_2_DS_2_-VA score. While the applied PDC cut-off of 80% is widely accepted across studies, growing evidence supports a stricter adherence threshold of PDC ≥90%, which has been associated with a lower risk of stroke in some studies.^12,13^ Based on this evidence, we chose a threshold of PDC ≥90% for the sensitivity analysis which resulted in a marked drop in 3-year adherence rates to 78.8% in patients with a CHA_2_DS_2_-VA scores of ≥2.

Likewise, persistence appeared suboptimal even among high-risk patients.^4^ A fifth of these high-risk patients displayed gaps in therapy comprising ≥20% of the coverage, which may increase the risk of adverse events.^45^

Guidelines indicate that long-term therapy offers limited benefit in patients with CHA_2_DS_2_-VA scores of ≤1, which possibly explains the much lower persistence rates found in these patient groups.^4^

AF recurrence remains common despite successful ablation, with a meta-analysis including 1774 patients across 13 studies showing AF recurrence in more than 40% of patients within 62 months post-procedure.^46^ A significant proportion (≥15%) of these recurrences may be silent or asymptomatic.^47^ This might result in unintentional treatment gaps and leave patients unprotected, underlining the need for post-ablation follow-up and patient education among patients with life-long indications for DOAC therapy. In this study, we did not account for repeated ablations during the follow-up period, which may bias towards higher adherence, especially in low-risk patients, as repeated procedures entail repeated indication for DOAC therapy and increase patient awareness.

Most studies on DOAC adherence and persistence only provide insights into a fraction of the average time spent on active DOAC therapy, particularly given a mean age below 70 in our CHA_2_DS_2_-VA ≥2 group. Across decades of treatment, compliance would expectedly continue to decline.^17^ At 5 years, persistence is reported around 60% across a general AF population in Western countries.^14^ As patients age and comorbidities arise, the thromboembolic risk increase, as does the risk for potential intolerable side effects of DOAC therapy. This may further impact compliance. LAAC could be considered an emerging alternative for stroke prevention that circumvents the problem of decade-long compliance and tolerance as outlined in the recent overview provided by Potpara *et al.* Europace 2025.^18^

In the recent OPTION-trial, LAAC showed the same stroke prevention efficacy over 3 years compared to OAC in the post-ablation setting, while the hazard ratio for non-procedural major bleeding or clinically significant non-major bleeding was 0.44 (95% CI, 0.33–0.59).^41^ Importantly, it was demonstrated that concomitant procedures combining LAAC and PVI are safe and feasible.

This study assumes that prescribers follow current guidelines. Nevertheless, education, both of healthcare professionals and patients has the potential to further improve care in AF patients. The STEEER-AF trial demonstrated that a structured educational programme for healthcare professionals improved adherence to guideline-directed AF management, and the AF-EduCare trial similarly showed the value of targeted patient education in reinforcing medication adherence and awareness of stroke risk.^48,49^

The low incidence of thromboembolic outcomes in this study was consistent with previous cohort studies and meta-analyses on AF patients undergoing catheter ablation.^50,51^ Whether AF ablation alters the ischaemic risk or these findings reflect the lower risk among patients undergoing AF ablation is unclear. However, the stroke rates in this study were lower than what was observed in the EAST-AFNET 4 Trial on early rhythm control and anticoagulation, with a lower AF burden following ablation being a likely mechanism.^39,52–54^ While DOAC cessation post-ablation may appear safe in selected patients at lower risk, further evidence is needed, particularly in higher-risk populations.^50,55–58^ In contrast to the current dichotomous approach, quantification and monitoring of post-ablation residual AF burden could refine stroke risk stratification. Efforts have been made by the European Society of Cardiology Council on Stroke and the European Heart Rhythm Association to develop a novel unifying definition of AF burden that may help identify patients in whom DOAC therapy is most critical.^59^ With this approach, cessation of DOAC therapy may also be considered as an informed choice in patients with low-risk AF phenotypes.^60^ Nevertheless, careful selection and shared-decision making on stroke prevention strategy after AF ablation is underscored by the recent evidence investigating DOAC continuation, cessation, or LAAC in this setting.

Furthermore, our findings must be interpreted in light of the Danish healthcare setting.^19^ Previous studies have shown that countries with a strong social welfare system and drug reimbursement policies tend to have higher adherence rates for chronic therapies, including oral anticoagulants.^14,61^

### Strengths and limitations

The inclusion of an unselected, nationwide cohort of all patients undergoing AF ablation who initiated DOAC therapy supported generalizability. The DNPR provides longitudinal nationwide coverage with generally high PPVs for key cardiovascular diagnoses used in this study, as previously described.^21–23,25,26,29,62^ The Danish Civil Personal Registration System enabled complete follow-up for all patients.

Several limitations must be acknowledged. Firstly, the rationale behind treatment discontinuation or interruption remains unknown. While most instances of non-persistence may reflect discontinuation of therapy, some may reflect clinical decisions, patient preferences or other unrecorded factors. Second, like all prescription registry studies, our analysis relied on drug dispensing as a proxy for medication intake. Thus, adherence measured as PDC reflects drug availability rather than usage, leading to potential overestimation of true adherence as we cannot account for patient factors leading to non-ingestion of available medication. Third, since the analyses were anchored at the date of ablation rather than the date of AF diagnosis, we were unable to account for medication stockpiling prior to the procedure, which could lead to adherence underestimation.

## Conclusion

While adherence to DOAC therapy was acceptable in patients with CHA_2_DS_2_-VA ≥2, persistence declined over time and more than one quarter of these patients had interrupted treatment at 3 years. Further evaluation of current strategies to ensure effective long-term stroke prevention in this population seem warranted.

## Supplementary Material

euag151_Supplementary_Data

## Data Availability

The data underlying this study are derived from Danish nationwide health registries and cannot be shared publicly or with third parties due to data protection regulations.
