# Supplementary material for: Compliance to direct oral anticoagulation therapy and clinical outcomes after catheter ablation for atrial fibrillation: a nationwide cohort study
Source: Europace. 2026 Jun 30;28(6):euag151. doi: 10.1093/europace/euag151 (PMC13318155; doi:10.1093/europace/euag151)
Supplement: euag151_Supplementary_Data [file euag151_supplementary_data.docx]

**Supplementary material**

**Supplementary table 1:** ICD-10, NOMESCO and ATC-codes for clinical events and baseline covariates

| ***Clinical events*** | ***ICD-10 code(s)*** | ***PPV*** | ***Cohort validated*** |
| --- | --- | --- | --- |
| Ischemic stroke | DI63  DI64  DI678A  DI678B | 93.5 (82.5-97.8) | Admission to neurological ward, 2010^1^ |
| Transient ischemic attack | DG458  DG459 | 60.5 (52.0-68.3) | 1994-1999^2^ |
| Systemic embolism | DI74* (excl. DI742B, DI743B) |  | Not validated |
| Intracranial hemorrhage | DI60  DI61  DI62 | 92.9 (87.9-95.9) | 2019, age ≥65^3^ |
| Extracranial major bleeding | DD62§  DD500§  DI85§  DI86A§  DJ942  DK25* (excl. DK253, DK257, DK259)  DK26* (excl. DK263, DK267, DK269)  DK27* (excl. DK273, DK277, DK279)  DK28* (excl. DK283, DK287, DK289)  DK290  DK298A  DK625  DK920  DK921  DK922  DN02§  DR04  DR31  BOQA00-BOQA05^a^§ | 94.1 (92.3-95.4) | 2019, Age ≥ 65y^3^ |
| ***Baseline characteristics*** | ***ICD-10 code(s) and NOMESCO codes*** |  |  |
| Atrial fibrillation | DI48 | 94.9 (88.5-97.8) | 2010-2012^4^ |
| Radiofrequency ablation | BFFB04 | 100 (91-100) | 2016, age 60-80^5^ |
| Congestive heart failure | DI50*  DI110  DI130  DI132 | 82.4 (73.8-88.9) | 2017-2022^6^ |
| Hypertension | DI10  DI11  DI12  DI13  DI15 | 90.0 (89.7-90.4) | 2005-2017^7^ |
| ***Hypertension (continued) ^†^*** | ***ATC code(s)*** |  |  |
|  | C02A  C02B  C02C  C02D  C02L  C03A  C03B  C03D  C03E  C03X  C04A  C05  C07  C09 | 93% | Different lookback period than validation study.^7^ |
| Diabetes | DE10  DE11  DE12§  DE13§  DE14§  DO24 (excl. DO244) §  DG632§  DH360§  DN083§ | 96.0 (86.3-99-5) | 1998-2007 ^8^ |
| ***Diabetes (continued) ^‡^*** | ***ATC code(s)*** |  |  |
|  | A10A§  A10B§  A10BA02§ |  | Not validated |
| Vascular disease | DI21  DI23  DI24§  DI25§  DI70  DI71  DI739  DI74 | 98% (89.4-99.9) 100% (92.9-100) | 1998-2007 ^8^ |
| Abnormal kidney function | DN17  DN18  DN19  DI12  DI13 | 100% (92.9-100) | 1998-2007^8^ |
| Abnormal liver function | DK70*  DK71  DK72§  DK73  DK74  DK75§  DK76  DI982§  DI85§ | 100% (92.9-100) | 1998-2007^8^ |
| Alcohol | DI426  DK292  DK70*  DF101  DF102 |  | Not validated |

*******Including all subcodes
***^†^***Hypertension defined by simultaneous use of ≥2 antihypertensives in combination
***^‡^***Diabetes defined by use of blood-glucose lowering agents, excl. the use of metformin in women of fertile age (<40)
§Not included in cited validation study

**Supplementary references**

1. Wildenschild C, Mehnert F, Thomsen RW, et al. Registration of acute stroke: validity in the Danish Stroke Registry and the Danish National Registry of Patients. *Clin Epidemiol*. 2014;6:27-36. doi:10.2147/clep.S50449

2. Johnsen SP, Overvad K, Sørensen HT, Tjønneland A, Husted SE. Predictive value of stroke and transient ischemic attack discharge diagnoses in The Danish National Registry of Patients. *J Clin Epidemiol*. Jun 2002;55(6):602-7. doi:10.1016/s0895-4356(02)00391-8

3. Thaarup M, Nielsen PB, Olesen AE, et al. Positive Predictive Value of Non-Traumatic Bleeding Diagnoses in the Danish National Patient Register. *Clin Epidemiol*. 2023;15:493-502. doi:10.2147/clep.S400834

4. Sundbøll J, Adelborg K, Munch T, et al. Positive predictive value of cardiovascular diagnoses in the Danish National Patient Registry: a validation study. *BMJ Open*. Nov 18 2016;6(11):e012832. doi:10.1136/bmjopen-2016-012832

5. Adelborg K, Sundbøll J, Munch T, et al. Positive predictive value of cardiac examination, procedure and surgery codes in the Danish National Patient Registry: a population-based validation study. *BMJ Open*. Dec 9 2016;6(12):e012817. doi:10.1136/bmjopen-2016-012817

6. Bonnesen K, Witt CT, Løgstrup B, Eiskjær H, Schmidt M. Validity of heart failure diagnoses, treatments, and readmissions in the Danish National Patient Registry. *Int J Popul Data Sci*. 2024;9(1):2394. doi:10.23889/ijpds.v6i1.2394

7. Bonnesen K, Schmidt M. Validity of Prescription-Defined and Hospital-Diagnosed Hypertension Compared with Self-Reported Hypertension in Denmark. *Clin Epidemiol*. 2024;16:249-256. doi:10.2147/clep.S448347

8. Thygesen SK, Christiansen CF, Christensen S, Lash TL, Sørensen HT. The predictive value of ICD-10 diagnostic coding used to assess Charlson comorbidity index conditions in the population-based Danish National Registry of Patients. *BMC Med Res Methodol*. May 28 2011;11:83. doi:10.1186/1471-2288-11-83
